# Molecular Mechanisms of Trastuzumab Resistance in HER2 Overexpressing Breast Cancer

**DOI:** 10.4061/2011/352182

**Published:** 2011-09-06

**Authors:** Gabriel L. Fiszman, María A. Jasnis

**Affiliations:** Immunobiology Department, Institute of Oncology A. H. Roffo, University of Buenos Aires, Avenida San Martín 5481, CP1417 DTB Buenos Aires, Argentina

## Abstract

The epidermal growth factor receptor 2 (HER2) is a tyrosine kinase overexpressed in nearly 20% to 25% of invasive breast cancers. Trastuzumab is a humanized monoclonal antibody that targets HER2. The majority of patients with metastatic breast cancer initially respond to trastuzumab, however, within 1 year of treatment disease progresses. Several molecular mechanisms have been described as contributing to the development of trastuzumab resistance. They could be grouped as impaired access of trastuzumab to HER2, upregulation of HER2 downstream signaling pathways, signaling of alternative pathways, and impaired immune antitumor mechanisms. However, since many of them have overlapping effects, it would be of great clinical impact to identify the principal signaling pathways involved in drug resistance. Significant efforts are being applied to find other therapeutic modalities besides trastuzumab treatment to be used alone or in combination with current modalities.

## 1. Introduction

Breast cancer is the most common malignancy worldwide and the second leading cause of cancer death in women in USA and the first one in Argentina [1]. In the past decades, the development of strategies for breast cancer treatment focused on understanding the expression, regulation, and function of critical signaling pathways involved in cancer initiation and progression and allowed the identification of breast cancer subsets with different biology [2]. One of the most important targeted therapies has been the use of the antihuman epidermal growth factor receptor 2 (HER2) for tumors overexpressing this receptor [3].

Breast cancer is a heterogeneous disease that can be classified in different subsets with distinct biology and molecular profiles [4], some of which can be associated with enhanced tumor aggressiveness and poor clinical outcome [5]. 

Breast tumors vary according to the expression of estrogen receptor (ER), progesterone receptor (PR), and amplification of HER2 which is overexpressed in approximately 20% to 25% of invasive breast cancers [6]. The resulting subgroups are important not only for clinical behaviour and prognosis, but also for predictive response to targeted therapies against these receptors and the pathways they activate.

The HER2/neu gene encodes a 185-kDA transmembrane tyrosine kinase (TK) receptor that belongs to the EGF receptor (EGFR) family which consists of EGFR/ErbB1 (HER1), ErbB2 (HER2), ErbB3 (HER3), and ErbB4 (HER4). All receptors, with the exception of HER3, contain a cytoplasmatic TK region and all, with the exception of HER2, bind specific ligands via extracellular domains. Upon ligand binding, receptors dimerize using HER2 as their preferential binding partner [6]. These receptors are expressed in a variety of tissues of epithelial, mesenchymal, and neuronal origin [7]. Activation of the HER receptors under physiological conditions is controlled by spatial and temporal expression of their ligands, members of the EGF family of growth factors. Homo- or heterodimerization of receptors after ligand binding results in the phosphorylation of residues from the intracellular domain of the receptor, resulting in the recruitment of signaling molecules from the cytoplasm and the initiation of several signaling pathways. HER2 dimerization triggers diverse cellular processes related to enhanced cell motility, survival, and proliferation as well as resistance to apoptosis [8]. Certain pathways are preferentially modulated by different receptors due to the ability of each receptor to bind specific effector proteins. Two of the main activated pathways are RAS/Raf/MAPK and the phosphatidylinositol 3-kinase (PI3K)–Akt pathways [9].

Overexpression of HER2 enables constitutive activation of growth factor signaling pathways, serving as oncogenic drivers in breast cancer. It is well known that cancer patients whose tumors have alterations in HER receptors tend to have a more aggressive disease associated with predictive factors of a poor clinical outcome [10]. Constitutive EGFR activation can be elicited by EGF-related growth factors produced either by the tumor cells themselves or by the surrounding stromal cells [11].

## 2. HER2 as Target for Cancer Therapy

Amplification of HER2 was originally detected in a subset of breast tumours [12] as well as in gastric [13], ovarian [14], and salivary gland cancers [15]. Concerning breast cancer treatment, HER2 has features that allow it to be considered as an ideal therapeutic target, since their levels strongly correlate with tumorigenesis, as demonstrated by gain [16] and loss of function [17], gene overexpression in metastatic breast cancers [12], and its association with poor disease-free survival [18]. Overexpression (25-fold increase in HER2 gene copy numbers) is found in nearly 30% of breast cancer [19] either in primary tumors as well as in metastatic sites, indicating that anti-HER2 therapy would be an effective approach for all disease sites. Hence, targeting HER2 protein could reduce the pathogenicity caused by tumor cells [20].

Two important types of HER2 inhibitors are currently in clinical use: humanized antibodies directed against EGFR/ErbB2 and small-molecule tyrosine-kinase inhibitors (TKIs) that compete with ATP in the tyrosine-kinase domain of the receptor. In preclinical models, both inhibitors rapidly downregulate PI3-AKT, MAPK, SRC, and STAT signaling, consequently blocking the proliferation of tumour cells and human xenografts in nude mice [21, 22].

Antibody-based therapy targeting HER2 is based on the development of monoclonal antibodies (mAbs) against epitopes present in the HER2 extracellular domain. Upon binding to their cognate epitopes, these antibodies exert their antitumor effects by multiple mechanisms.

Trastuzumab (Herceptin, Genentech Inc., San Francisco Calif, USA) is a humanized recombinant mAb that binds to the extracellular domain of the human HER2 protein [23]. Currently, it is the only HER2-targeted therapy approved by the FDA for metastatic breast cancer treatment. It selectively exerts the antitumor effects in experimental animals and patients with HER2-amplified breast tumors but not in tumors with normal HER2 expression [24, 25]. However, the clinical efficacy of trastuzumab is limited, since a significant number of patients with HER2 overexpressing tumors will be initially or eventually resistant to trastuzumab [26]. An overall response rate (complete + partial responses) ranging from 15% to 30% has been obtained when used as single agent; however, higher response rates (50%–80%) have been reported when used in combination with standard chemotherapy for metastatic disease [27–29].

Understanding the mechanisms of resistance to trastuzumab is, therefore, crucial for the development of new therapeutic strategies [7].

## 3. Mechanisms of Trastuzumab Action

Although trastuzumab is currently widely used for the treatment of HER2-overexpressing breast cancer, its mechanism of action is not fully understood [30].

### 3.1. Effect on Cell Cycle

Trastuzumab binds to HER2 with high affinity, inducing a cytostatic effect associated with G1 arrest, reduction in cell proliferation, and apoptosis via upregulation of the cyclin-dependent kinase inhibitor p27^kip1^ [31]. It has been demonstrated by Western blot and by immunoprecipitation that breast tumor cell lines treated in vitro with trastuzumab increased p27^kip1^ levels and interaction with CDK2, resulting in a decrease in CDK2 activity [32].

### 3.2. Effect on the PI3K Pathway

HER2 and EGFR activate signaling through PI3K/Akt pathway. When PI3K becomes activated, it generates phosphoinositides which cause AKT translocation to the plasma membrane, where it phosphorylates and becomes able to phosphorylate different targets. On the other hand, AKT can be negatively regulated by antagonizing the action of the phosphatase and tensin homologue (PTEN) on PI3K. Trastuzumab can possible reduce the signaling by this pathway, promoting arrest of cell proliferation and apoptosis [33].

### 3.3. Inhibition of HER2 Extracellular Domain Proteolysis

Overexpressed HER2 undergoes proteolytic cleavage, releasing the extracellular domain which generates a truncated membrane-bound fragment (p95) found in serum patients [34]. Molina et al. [35] demonstrated that trastuzumab inhibited HER2 cleavage and thus the shedding of the extracellular domain, possibly by the inhibition of metalloproteinases activity. Indeed, the authors detected a phosphorylated truncated receptor in almost half of the human breast cancer serum samples. A decrease in serum HER2 extracellular domain upon trastuzumab treatment could be considered as predictor of host response [36], since it was associated with improved progression-free survival.

### 3.4. Inhibition of Angiogenesis

Trastuzumab modulates the effects of different pro- and anti-angiogenic factors as well as normalization and regression of the vasculature in a xenograft model of human HER2-overexpressing breast tumor. A reduction of VEGF production only in vitro has been described, indicating that in vivo, the effect of trastuzumab is VEGF-independent [37]. A decrease in microvessel density in vivo has also been observed [38]. Expression of proangiogenic factors was reduced, while expression of antiangiogenic factors was increased in trastuzumab treated tumor-bearing animals versus controls.

### 3.5. Internalization and Degradation of HER2 Protein

Another mechanism of trastuzumab effect can be due to downregulation of HER2 through endocytosis; clinical data confirming this pathway is conflicting. Hommelgaard et al. [39] using the breast cancer cell line SKBR3 described the preferential association of HER2 with the plasma membrane protrusions, making HER2 an internalization-resistant receptor. Cuello et al. [40], using the same tumor cell line, reported that trastuzumab downregulated HER2 protein. The formation of large lattices between antibodies and antigens at cell surface, collapsing into the cytoplasm and being degraded in lysosomes has been proposed [41]. In this case, internalization and degradation would be increased by the concomitant use of antibodies targeting different epitopes on HER2, as shown by Portera et al. [42].

### 3.6. Immune-Mediated Response

Data from in vivo experiments and clinical trials indicate that the efficacy of trastuzumab could be partially related to its induction of an immune response. In vivo breast cancer models and clinical trials have demonstrated that trastuzumab has not only cytostatic but also cytotoxic properties, at least in part via antibody-dependent cellular cytotoxicity (ADCC). This immunological effect has been observed in vitro with a variety of breast cancer cell lines [43]. Stockmeyer et al. [44] demonstrated that apoptosis and ADCC against the breast cancer cell line SKBR3 treated with bispecific anti-HER2 mAb only happened when neutrophils were present.

It is known that ADCC is mainly due to natural killer cells (NK) that, when activated, express the Fc*γ* receptor that binds the Fc domain of the IgG1 trastuzumab. In a xenograft model, activated NK could lyse tumor cells associated with a tumour regression rate of 96%. In mice lacking the Fc receptor, only 29% tumour growth inhibition was observed [44]. These results led to the conclusion that an active immune response to trastuzumab may be responsible for the antitumor activity. Gennari et al. [45] selected 11 patients with HER2-positive breast tumours receiving trastuzumab at a standard dose before breast surgery. Trastuzumab treated patients who showed complete or partial remission were found to have a higher in situ infiltration of leukocytes and a higher capability to mediate in vitro ADCC. However, they could not detect changes in total HER2 levels or downstream signalings such as alterations in cell proliferation or tumor vasculature. It is necessary to continue studying the participation of ADCC in mediating the response to trastuzumab.

### 3.7. Inhibition of DNA Repair

Pietras et al. [46] observed that trastuzumab partially inhibited DNA adducts repair induced by cisplatin and that it also blocked DNA synthesis after radiation in in vitro studies [47].

## 4. Mechanisms of Trastuzumab Resistance

It should be noted that most of the mechanisms of trastuzumab resistance have been identified in preclinical models and have not been completely validated in clinical samples. One important goal is to clarify which of all the mechanisms are clinically relevant. However, it is known that, as with other drugs, clinical resistance will be multifactorial. [7, 30, 48]. 

**Figure 1:**
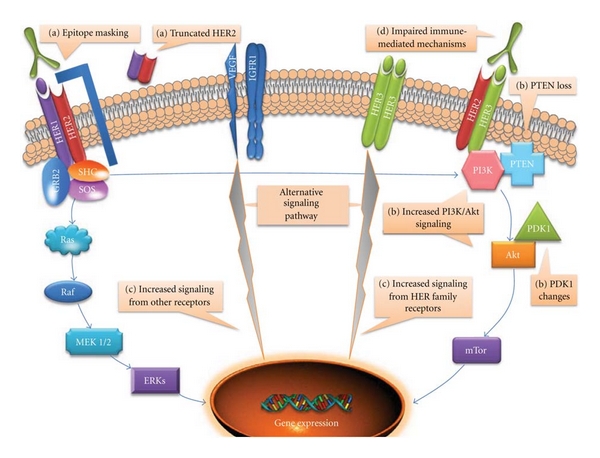
Relevant mechanisms of trastuzumab resistance in breast cancer: (a) Impaired trastuzumab binding to HER2: truncated HER2 and epitope masking. (b) Upregulation of HER2 downstream signaling pathways: PTEN loss, increased PI3K/Akt activity and PDK1 changes. (c) Alternative signaling pathways: Increased signaling from HER family and other receptors. (d) Impaired immune-mediated mechanisms.

The most relevant mechanisms of trastuzumab resistance in breast cancer are shown in Figure 1.

## 5. Impaired Trastuzumab Binding to HER2

### 5.1. Truncated HER2

Different metalloproteases cleave the extracellular domain of HER2, creating a truncated receptor to which trastuzumab is unable to bind [49]. Thus, the membrane-bound portion, remains as a truncated constitutively active HER2 receptor with kinase activity but lacking the extracellular domain to bind trastuzumab [50]. However, in vitro data suggested that trastuzumab could block the cleavage of HER2 and consequently the production of constitutively active p95HER2 [35]. A retrospective clinical study revealed a strong association between the presence of p95HER2 and patients resistance to trastuzumab treatment [50]. Extracellular domain HER2 level has been evaluated as potential predictor for treatment response in metastatic breast cancer but had low predictive values for clinical benefit with trastuzumab therapy [51]. According node status and HER2 expression, Molina et al. [49] reported that the expression of p95 HER2 was more frequent in node-positive cases compared to node-negative ones.

### 5.2. Masking with MUC 4 and CD44/Hyaluronan Polymer Complex

Epitope masking has also been described as a mechanism of resistance to trastuzumab. Mucin 4 (MUC4) is membrane-associated glycoprotein [52], which consists of several highly glycosylated proteins that form protective barriers on epithelial cells including mammary epithelium. In this sense, MUC4 can contribute to cancer progression due to its ability to inhibit recognition of cancer cells by the immune system, promoting tumor progression, metastasis, and suppression of apoptosis [53, 54]. MUC4 may interfere with trastuzumab binding by masking HER2 receptor [55, 56]. In a preclinical study with the human HER2-positive trastuzumab-resistant JIMT-1 cell line, expression of MUC4 was associated with decreased antibody-binding capacity, being able to reverse resistance using RNA interference to knockdown MUC4 [56].

Another HER2 masking that blocks trastuzumab binding is mediated by CD44, a transmembrane receptor for hyaluronan. It has been shown that binding of polymeric hyaluronan, activates CD44-mediated signaling pathways including RAS and PI3K [57]. Binding of endogenous hyaluronan polymer to CD44 contributes to PI3K/Akt activation [58]. Blocking CD44- hyaluronan polymer binding by the use of anti-CD44 antibodies or hyaluronan oligomers suppressed the PI3K/Akt signaling pathway, inhibiting anchorage-independent tumor cell growth.

## 6. Upregulation of HER2 Downstream Signaling Pathways

### 6.1. PTEN Loss

The loss of function of PTEN (phosphatase and tensin homolog) caused by mutation, or by transcriptional regulation, has been described in several tumors including nearly 50% of breast cancers [59]. PTEN normally inhibits the activation of PI3K; therefore, PTEN loss results in constitutive activation of PI3K/Akt [60]. Decreased expression or activity of PTEN blocked trastuzumab-mediated growth inhibition in HER2 overexpressing breast cancer cells [61]. Nagata et al. [33] have emphasized the important role of decreased expression of PTEN protein resulting in an increase in PI3K/Akt phosphorylation and signaling, preventing trastuzumab-mediated growth arrest of HER2-overexpressing breast cancer cells. Patients with PTEN-deficient HER2-overexpressing metastatic breast cancer had significantly worse responses to trastuzumab than those with normal PTEN tumors [33, 59]. Berns et al. [62], using a large-scale RNA interference screen, identified PTEN as the only gene whose knockdown resulted in trastuzumab resistance.

In clinical trials, Dave et al. [63] confirmed that low PTEN or activating mutation in PIK3CA conferred resistance to trastuzumab regimen, whereas low PTEN tumors were associated with a high pathologic complete response. Activation of PI3K pathway was associated with trastuzumab resistance, whereas low PTEN predicted for response to lapatinib. These observations support clinical trials with the combination of both agents.

### 6.2. Increased PI3K/Akt Activity

PI3K mutations are also involved in trastuzumab resistance through PI3K/Akt pathway activation. The *PIK3R1* gene encodes the PI3K regulatory subunit p85*α*, affecting its function and inducing constitutive activation of the PI3K/Akt pathway [64, 65]. In addition, *PIK3CA *(activator of the same pathway and frequently mutated in breast cancer) is the gene that encodes the catalytic subunit of p100*α* of PI3K. It is frequently mutated or overexpressed in human cancer [66] and is able to confer resistance to trastuzumab in cell culture [66]. In a clinical study using 55 patients with breast cancer, Berns et al. [62] described significantly improved ability to detect patients with low response to trastuzumab when combining the analysis of low PTEN expression with the presence of oncogenic PIK3CA mutations. They also described an association of PIK3CA hotspot mutations and reduced time to progression after therapy.

Junttila et al. [67] showed that trastuzumab disrupts ligand-independent ErbB2/ErbB3/PI3K complexes blocking AKT signaling; when PI3K is mutated, complex disruption does not inhibit AKT, explaining why trastuzumab is ineffective in some tumors. Recent studies using HER2-amplified breast cancer cell lines showed that those cell lines with PIK3CA hotspot mutations were significantly more resistant to trastuzumab than those without mutations [68]. Thus, assessment of PI3K pathway activation might be considered a biomarker to identify patients unlikely to respond to trastuzumab-based therapy. An activating mutation of Akt1 situated in the pleckstrin homology domain (E17K) was identified in breast cancer and occurs early in the development of the disease [69]. However, this mutation has not been identified in HER2-overexpressing tumors [70].

### 6.3. PDK1 Changes

Overexpression of PDK1 was found in approximately 20% of breast cancers [71]. Tseng et al. [72] in a preclinical model reported that the combined use of PDK-inhibitors with trastuzumab reversed the trastuzumab-resistant phenotype of SKBR3 human breast cancer cells.

### 6.4. Modulation of p27^Kip1^

Another mechanism of trastuzumab resistance is dependent of the cyclin-dependent kinase inhibitor p27^kip1^ [73]. Several studies have suggested that p27^kip1^ is a critical element of trastuzumab response and that its downregulation can promote trastuzumab resistance [74]. The growth inhibitory properties of trastuzumab depend, in part, upon the effects on the cyclin-dependent kinase-inhibiting protein p27^kip1^ [75]. Treatment with cyclin-dependent kinase 2 (CDK2) inhibitors led to a dramatic decrease in cell proliferation and enhanced apoptosis [76]. In vivo, CDK2 inhibition significantly reduced tumor growth of trastuzumab-resistant xenografts. They suggested that the use of CDK2 inhibitors in patients with breast tumors with HER2 and cyclin E coamplification/overexpression might be a valid strategy. Le et al. [77] have also suggested that any drugs or therapies that directly affects p27^kip1^ or its signaling would influence the efficacy of trastuzumab. Actions on more than one target or pathways may have synergistic or additive effect against overexpressing HER tumor cells.

## 7. Alternative Signaling Pathways

Signaling through multiple alternative pathways has been linked to trastuzumab resistance in various preclinical models.

### 7.1. Increased Signaling from HER Family Receptors

Compensatory crosstalk occurs among receptors within the HER family [78–80]. Although trastuzumab reduces HER2-mediated signaling, it might not reduce signaling from other HER receptors. HER1/HER3 heterodimers or HER1/HER1 homodimers expressing cells might initiate MAPK and PI3K signaling even in the presence of trastuzumab [21, 78].

HER3 is the favored receptor for dimerization with HER2 [81], and growing evidence supports HER3 as being a required partner in HER2-overexpressing breast cancer [82, 83]. The oligomerization properties of the HER receptors confirmed that interactions between EGFR/HER2 and HER2/HER3 are detected in the presence of ligand. In addition, they found that trastuzumab is ineffective in blocking HER2/HER3 dimerization [84].

Transforming growth factor *α* (TGF *α*) is a ligand of the HER family, and it is involved in the formation of HER2 heterodimers. In vitro, trastuzumab was less efficient in inhibiting cell growth in presence of TGF *α* [85]. In addition, increased levels of the HER family ligands such as heregulin and EGF blocked trastuzumab-mediated growth inhibition in HER2 overexpressing breast cancer cell lines [86].

### 7.2. Increased Signaling from Other Receptors

#### 7.2.1. Insulin-Like Growth Factor-1 Receptor Overexpression

The insulin-like growth factor-1 receptor (IGF-1R) is a transmembrane TK receptor frequently expressed in human breast cancers. Its downstream effectors promote proliferation and metastatic dissemination [30]. HER-2 and the IGF-1R share common postreceptor signaling pathways. Cell proliferation is also stimulated by IGF-1R which interacts with HER2 in trastuzumab-resistant cell lines. The first research article that focused on molecular mechanisms contributing to trastuzumab resistance described IGF1R signaling as the contributing cause of resistance [87]. The authors demonstrated that trastuzumab-mediated growth inhibition was lost in breast cancer cells that overexpressed both HER2 and IGF1R. Preclinical studies have implicated IGF-1R-signaling in resistance to trastuzumab and anti-IGF-1R drugs restored sensitivity in resistant cells [88]. IGF-1R-mediated resistance to trastuzumab seems to involve the PI3K/Akt pathway, leading to enhanced degradation of p27 [48, 89]. It can be suggested that coexpressing HER2 and IGF-1R tumors are more likely to be resistant to trastuzumab therapy.

#### 7.2.2. Vascular Endothelial Growth Factor Overexpression

Increase in tumor angiogenesis in breast tumors is associated with overexpression of HER2 [90]. Mice treated with trastuzumab induce normalisation and regression of the vasculature in a xenograft model of HER2-overexpressing breast tumour. Izumi et al. [37], however, observed a reduction in vascular endothelial growth factor (VEGF) production by cancer cells only in vitro, indicating that a VEGF-independent antiangiogenic mechanism might act in vivo. This mechanism appears to be due to the modulation of different regulators of the complex machinery of angiogenesis.

#### 7.2.3. c-Met Coexpression

The c-Met receptor, another TK receptor, can be coexpressed with HER2 in cell lines contributing to trastuzumab resistance through sustained Akt activation. HER2-overexpressing breast cancer cells respond to trastuzumab with a rapid upregulation of c-Met receptor expression and activation protecting cells against trastuzumab [91]. Loss of c-Met function by RNA interference improves the response of these cell lines to trastuzumab. Met-amplification, and certain alternative signals from Met to HER3 have been found capable of inducing acquired resistance to the EGFR TKIs gefitinib and erlotinib in non-small cell lung cancer [92].

#### 7.2.4. Hormone Receptor Signaling

A potential mechanism of resistance is through ER (it says estrogen receptor) pathway. The existence of a potential crosstalk between HER2 and this pathway has been widely described [93–95]. Upregulation of the estrogen receptor after treatment with lapatinib (anti-HER2 mAb) suggests that the receptor might act as a redundant survival pathway [96].

Crosstalk between the steroid hormone receptors for ER and progesterone (PR) and the HER family appears to be a hallmark of breast cancer growth. Proietti et al. [97] highlighted signal transducer and activator of transcription 3 (Stat3) as a key protein activated by heregulin (HRG), a ligand of the HER receptors through co-opted ligand-independent PR function as a signaling molecule. Stat3 activation was an absolute requirement in HRG-induced mammary tumor growth, and targeting Stat3 effectively inhibited growth of breast cancer cells with activated HRG/HER2 and PR. A novel potential therapeutic intervention in PR- and HER2-positive breast tumors involving the specific blockage of PR signaling activity could be investigated.

Other hormones such as prolactin acting through Janus kinase activation can transactivate HER2, leading to cell proliferation even in the presence of trastuzumab [98].

#### 7.2.5. Cyclin E Amplification/Overexpression

The clinical relevance of cyclin E amplification/overexpression detected in numerous HER2 breast cancer patients has been reported [76], resulting in a lower clinical benefit rate and progression-free survival after trastuzumab therapy. High cyclin E expression has been proposed as a marker of poor clinical outcome in breast cancer [99]. Furthermore, it has been recently shown that cyclin E levels decrease upon HER2 downregulation, suggesting that HER2 regulates cyclin E function [100]. The role of cyclin E in trastuzumab resistance suggests that treatment with CDK inhibitors is a probable approach for patients whose tumors display cyclin E amplification/overexpression.

#### 7.2.6. Tumor Necrosis Factor *α* Transactivation

Rivas et al. [101] have shown that tumor necrosis factor *α* (TNF*α*) induces HER2 phosphorylation in breast cancer cells, which is mediated by c-Src activation. Moreover, TNF*α* promoted HER2/HER3 heterocomplex formation, Akt activation, and NF-*κ*B transcriptional activation. Inhibition of HER2 by addition of AG825, an EGFR/HER2-tyrosine kinase inhibitor, or knockdown of HER2 by RNA interference blocked TNF*α*-induced NFkB activation and cell proliferation. However, trastuzumab could not inhibit TNF*α* ability to promote breast cancer growth. Interestingly, TNF*α* is able to transactivate HER2 and use it as a mandatory downstream signaling molecule in the generation of mitogenic signals. As TNF*α* has been shown to be present in the tumor microenvironment of a significant proportion of human infiltrating breast cancers, it would have clinical implication in HER2-positive breast cancer treatment.

#### 7.2.7. Erythropoietin Coexpression

Erythropoietin (EPO) has long been known to be an important hematopoietic cytokine that regulates the survival, proliferation and differentiation of the erythroid progenitor cells in the bone marrow [102]. EPO-induced cell signaling via interaction with specific tyrosine-phosphorylated regions within the activated receptor for erythropoietin (EpoR), leads to activation of downstream signaling pathways, such as the MEK/Erk and PI3K/Akt pathways [103]. These downstream signaling pathways activated by EPO via EpoR overlap and/or interact with those activated by HER2.

Recently, Liang et al. [104] demonstrated that the EpoR is coexpressed with HER2 in a significant percentage of human breast tumor specimens and breast cancer cell lines. Exposure of these dual-positive breast cancer cells to recombinant human erythropoietin (rHuEPO), activated cell signaling. Concurrent cell treatment with rHuEPO and trastuzumab reduced the cell responsiveness to trastuzumab both in vitro and in vivo. They identified Jak2-mediated activation of Src and inactivation of PTEN as underlying mechanisms through which rHuEPO antagonizes trastuzumab-induced therapeutic effects. 

#### 7.2.8. Contribution of Rac1 Activity

Rac1, a Ras-like small GTPase, has been implicated in the control of cell growth and morphology and is believed to be associated with breast cancer progression. Dokmanovic et al. [105] using SKBR3 cells have reported that resistance to trastuzumab was associated with increased Rac1 activity, leading to significant cytoskeleton disorganization. Redution of Rac1 activity with a specific inhibitor, restored trastuzumab-mediated endocytic downregulation of HER2 and reduced extracellular signaling regulate kinase activity in resistant clones. In addition, the role of Rho GTPases as a treatment target has been recently reviewed by Menna et al. [106]. These results highlight Rac1 as a potential therapeutic target for the treatment of trastuzumab-resistant breast cancer.

## 8. Impaired Immune-Mediated Mechanisms

Breast tumor cells isolated from in vitro resistance to trastuzumab remain sensitive to trastuzumab antitumor effects in vivo and to ADCC killing. Tumor cells resistant to trastuzumab in vitro, remain sensitive to its antitumor effect in vivo as well as to antibody dependant cellular cytotoxicity (ADCC) killing [107]. Gene polymorphism producing the phenotype expression of valine (V) or phenylalanine (F) at amino acid 158 on the Fc*γ*RIIIa modifies the affinity of IgG1 to the Fc*γ* receptor [108]. Immune effector cells carrying the Fc*γ*RIIIa V/V alleles mediate a stronger ADCC of trastuzumab than cells bearing the F allele [109]. In the clinic, the Fc*γ*RIII 158V/F polymorphism interferes with the ability to generate in vitro ADCC responses during trastuzumab treatment, impairing the clinical response and progression-free survival of patients in the metastatic setting [110]. Further evidence on the importance of ADCC was provided by Barok et al. [111], who showed that trastuzumab can trigger ADCC and destroy trastuzumab-resistant HER2 positive cell lines *in vitro* and *in vivo* xenografts. 

In summary, these observations point to ADCC as a major player in trastuzumab antitumoral effect. It would be very interesting to know how ADCC affects cells that are prone to enhance PI3K/Akt and RAS/Raf/MAPK pathways in order to survive. Multiple proteases involved with infection, inflammation, and tumor environments are able to cleave IgG molecules on site, generating monovalent or bivalent antibody fragments, Fab′ or F(ab′)2, which lack Fc-mediated effector function [112]. However, these antibody fragments can still bind to their cognate antigen, but lack binding sites for complement and for Fc receptors, and can no longer trigger Fc-mediated functions such as ADCC.

In our laboratory, we are studying the mechanisms involved in trastuzumab effect and resistance in different human breast cancer cells overexpressing HER2. Our study is mainy focused using 3D cultures of tumor cells (spheroids) in presence or not of syngeneic macrophages or peripheral mononuclear cells [113, 114].

## 9. Conclusion

Trastuzumab is an example of targeted therapy that offers significant clinical benefit in patients with HER2 overexpressing cancers in the adjuvant and metastatic settings. However, nearly 70% of patients, who initially reasonable option trastuzumab, demonstrate disease progression within 1 year of treatment. Elucidation of the mechanisms involved in *de novo* and acquired resistance to trastuzumab seem to be difficult due to the multiple mechanisms of action. A strategy to overcome resistance may involve the use of treatments that do not depend on molecular binding to the HER2 extracellular domain, including the use of TK inhibitors such as lapatinib or of inhibitors of the downstream HER2 signaling pathways. Another reasonable option would be to increase tumor cell destruction through extracellular mechanisms by increasing or enabling ADCC or by using immunotoxins such as the antibody-drug conjugate trastuzumab-DM1 [115] or the bispecific anti-HER2/CD3 Ab ertumaxomab. 

Based on emerging knowledge of the HER2-signaling pathways and of signaling through alternative pathways, several drugs against HER2-overexpressing breast cancers are in different phases of clinical development, whereas others are still struggling to leave the bench.

It will be of great clinical impact to discover and identify potential molecular predictors of response to current and new HER2-targeted agents. Understanding the mechanisms by which HER2 can be activated by nonclassical receptors and ligands is relevant in order not only to design new therapeutic approaches, but also to predict patient's response.

Many new agents are currently in preclinical stages of development. They include HER2 targeting agents as mAbs, TKIs, and vaccines. Pertuzumab, a recombinant humanized HER mAb that blocks dimerization of HER2 with HER3 and EGFR [116], is one of the new classes of drugs called dimerization inhibitors. It has the potential to block signaling by other HER family receptors as well as inhibiting signaling in cells that express normal levels of HER.
